# Electrocardiographic Responses Following Live-Fire Firefighting Drills

**DOI:** 10.1097/JOM.0000000000001730

**Published:** 2019-12

**Authors:** Denise L. Smith, Gavin P. Horn, Bo Fernhall, Richard M. Kesler, Kenneth W. Fent, Stephen Kerber, Thomas W. Rowland

**Affiliations:** Department of Health and Human Physiological Sciences, Skidmore College, Saratoga Springs, New York; Illinois Fire Service Institute; Illinois Fire Service Institute; Department of Mechanical Science and Engineering, University of Illinois, Urbana-Champaign; Department of Kinesiology & Nutrition, Integrative Physiology Laboratory, University of Illinois at Chicago, Chicago, Illinois; Illinois Fire Service Institute; Division of Surveillance, Hazard Evaluations, and Field Studies, National Institute for Occupational Safety and Health (NIOSH), Ohio; Firefighter Safety Research Institute, Underwriters Laboratories (UL), Columbia, Maryland; Department of Health and Human Physiological Sciences, Skidmore College, Saratoga Springs, New York

**Keywords:** arrhythmias, cardiac events, fire fighter, Ischemia

## Abstract

**Objective:**

Firefighting-related environmental and physiological factors associated with cardiovascular strain may promote arrhythmias and myocardial ischemia, which induce sudden cardiac events (SCE) in susceptible individuals. The present study evaluated electrocardiographic (ECG) changes that may reflect increased SCE risk following simulated live-firefighting.

**Methods:**

Using a repeated measures design, ECG tracings from 32 firefighters were recorded 12-hours post-firefighting in a residential structure and compared with a 12-hour control period.

**Results:**

Ventricular Ventricular arrhythmias were present in 20%, and ST segment changes indicative of myocardial ischemia in 16%, of firefighters 12-hours post-firefighting that were not detected in the control period.

**Conclusion:**

Live-firefighting induces significant ECG changes that include ventricular arrhythmias and ST segment changes, which may reflect myocardial ischemia. The implications of such ECG changes explaining increased cardiovascular risk in firefighters warrants further research.

Active firefighting is associated with a significant increased risk of sudden cardiac death.^1–4^ This increased risk is presumed to reflect the combined effects of increased physical work, psychological stimulation, exposure to particulate matter, and other products of combustion, hyperthermia, and dehydration during firefighting, all of which may augment arrhythmogenic sympathetic drive and collectively serve as cardiac stressors.^5^ Silent morbidities such as occult coronary artery disease and systemic hypertension, as well as obesity and poor cardiorespiratory fitness, may contribute to this predisposition.^6^

The validity of the assumption that the stresses of firefighting are responsible for provoking cardiac events would be supported by evidence that electrocardiographic (ECG) abnormalities indicative of ventricular tachyarrhythmias and myocardial ischemia are triggered during firefighting. The technical challenge of obtaining adequate ECG tracings during actual firefighting, however, has precluded substantial, direct insights. As feasible alternatives, ECG responses to a fire alarm,^7^ maximal treadmill exercise testing,^8^ and firefighting training sessions^9–11^ have been described. These studies have provided conflicting results, with some studies reporting ECG abnormalities indicative of myocardial ischemia and others demonstrating no significant ECG changes. Moreover, conclusions from these studies have been confounded by methodological weaknesses, including absence of a non-firefighting control group, variations in ECG criteria for indicating myocardial ischemia, and reliance on computer ECG analysis. It is uncertain, as well, whether ECG changes reported during or after firefighting reflect risk for cardiac death or are instead presumably-benign manifestations of sustained heart work (eg, as described in marathon running).^12^

The purpose of this study was to examine electrocardiographic tracings of firefighters for a 12-hour period following a simulated bout of live-fire active firefighting in comparison to findings in the same individuals during a control period in which individuals engaged in daily activities but did not undertake firefighting or other strenuous activities. Specifically, the goal was to test the hypothesis that firefighting activities create increased cardiac risk via arrhythmias and myocardial ischemia as manifest by electrocardiographic changes.

## METHODS

### General Procedures and Subject Characteristics

This study was conducted at the University of Illinois at Urbana Champaign, in collaboration with the First Responder Health and Safety Laboratory at Skidmore College, the National Institute of Occupational Safety and Health (NIOSH) and Underwriters Laboratories (UL) Firefighter Safety Research Institute (FSRI). IRB approval was acquired from the University of Illinois at Urbana-Champaign and NIOSH. Participants were recruited through a nationwide multimedia effort among individuals who teach and train at the Illinois Fire Service Institute’s (IFSI) Champaign campus. Inclusion criteria included being a fire fighter between the ages of 18 and 55 years, not being a smoker or other tobacco user, no known cardiovascular diseases or history of any neurological disorders, no history of abnormal digestive system complications including abnormalities with swallowing, esophageal or bowel strictures, gastrointestinal obstruction, or fistulas and do not use a pacemaker. Female participants were included after confirmation that they were neither pregnant nor lactating. All participants were required to complete a medical evaluation consistent with National Fire Protection Association (NFPA) 1582 within 12 months of study participation. Only experienced firefighters whose training was current and who were familiar with live-fire policies and procedures took part in the study. Standardized personal protective equipment (PPE) that was compliant with applicable NFPA standards was provided to study participants. Throughout the protocol, firefighters wore full PPE and self-contained breathing apparatus (SCBA).

Forty-one firefighters (37 men) from departments in Illinois, Georgia, Indiana, Ohio, South Dakota, and Wisconsin participated in this study. Due to issues with ECG data quality and one firefighter withdrawing prior to his first data collection session, the final sample for analysis included 32 firefighters. On average, these firefighters were 36.7±8.1 years old, 1.80±0.08m tall, weighed 88.3±15.4 kg, and had an average body mass index (BMI) of 27.1±3.4 kg/m^2^ with an average of 13.4±7.3 years of experience in the fire service.

### Firefighting Drills

As described fully elsewhere, participants completed a realistic firefighting response that involved a multiple-room fire (two separate, full-furnished bedrooms) in a 111m^2^ purpose built residential structure.^13^ The scenario was designed to more closely replicate typical fireground conditions found at a residential fire than is commonly replicated in training fire exercises that are often performed in a concrete building using only wood and straw as a fuel. Thus, firefighting was performed in a wood-framed residential structure that was finished with gypsum board. The structure also contained furnishings that are typically found in a residence to create a realistic fuel load and fire conditions that replicate typical fire scenarios. Briefly, common residential furniture was placed in the dining room and living room, which was not ignited by the fire, but which provided common obstacles to reaching the fires in the bedrooms. The bedrooms were furnished with beds, dressers, and a chair, and finished with bedding, curtains and wall hangings, and also contained a television and lamp. The exact same make and manufacture of furniture and furnishings were used in each replicate. Details of the thermal strain and chemical exposures experienced by the firefighter participants have been published elsewhere.^13,14^

Working in teams of two, firefighters performed typical firefighting operations such as incident command/pump operations, fire suppression, search and rescue, ventilation, and overhaul activities. The fire response scenarios lasted approximately 30minutes, including performing overhaul activities after the fire was suppressed. Once firefighters completed their job assignments, they were released to a “rehab” area, approximately 40m from the structure, to doff their gear. Firefighters remained in the rehab area for approximately 20 minutes where they cooled down and rehydrated. Once released from rehab, the firefighters showered and changed in to clean clothing, and ECG leads were attached (hair removal and skin prep occurred prior to firefighting activities to reduce delay in attaching leads after showering).

### Data Collection Period

Descriptive measures, including height and weight were obtained prior to testing using a digital scale (seca 284, Seca North America). ECG data were collected continuously for 12-hour periods following firefighting activity and during a control day using a portable ECG monitor (M12R Holter Recorder, Global Instrumentation, LLC). Electrodes were placed by trained personnel after showering. During the post fire suppression period, firefighters were released to a classroom space where they were free to engage in activities as they would at a fire station (though without additional fire responses or strenuous duty for the following 12 hours). Meals were provided at lunch and dinner time. Firefighters were instructed to perform typical activities on their control day and not to engage in firefighter, fire training, or strenuous activity. An off-duty day was chosen for the 12-hour control period. Electrodes were placed on the firefighters in the morning and they were asked to wear the monitor for at least 12 hours.

### ECG Analysis

ECG recordings were downloaded and processed using the Global Instruments Holter System Client (Global Instrumentation, LLC, Manlius, NY) using the arrhythmia and ST-episode detection program algorithms. A board-certified cardiologist (T.R.) reviewed and either confirmed or rejected the system-identified arrhythmias and ST-segment deviations. For the ST-segment review, 12-lead strips, obtained following firefighting and during the control day of all ST-segment elevations and depressions identified by the program as abnormal, were printed. ST segment changes considered consistent with myocardial ischemia were defined as a segment depression of more than or equal to 2mm which did not return to the baseline for more than or equal to 0.08seconds. Tracings in which similar configuration of the ST segment occurred with a depression of 1.0 to 2.0mm were considered to reflect possible myocardial ischemia. A depression of the J-point (junction of QRS complex and ST segment) with upward sloping of the ST segment that reached the baseline in less than 0.08seconds was considered physiologic.

A 12-lead strip, which was artifact free (baseline), was selected from each 12-hour control file and printed for analysis of the baseline electrocardiogram by the cardiologist. Analysis included heart rate, rhythm, intervals (P-R, QRS, QTc), axes (P, QRS, T, QRS-T Angle), ST-T wave changes, and signs of left ventricular hypertrophy.

### Data Analysis

Data were analyzed using IBM SPSS Statistics for Windows (Version 25, IBM Analytics, Armonk, NY). Data are reported as mean±standard deviation in tables and mean±standard error of the mean in figures. Ventricular and supraventricular ectopy were considered as dichotomous variables (“yes” or “no”) in statistical analyses. The McNemar test was used to determine whether the proportion of firefighters with detected ventricular or supraventricular ectopy differed between the 12-hour control and 12-hour post-firefighting activity periods. Tests were two-sided and statistical significance was taken at *P*<0.05.

## RESULTS

### Baseline Measures

Baseline ECG characteristics are reported in Table 1. Several non-normal ECG findings were observed on the baseline 12-lead electrocardiograms. One participant was found to have a short PR interval and QRS widening typical of ventricular pre-excitation (Wolff-Parkinson-White syndrome). Single participants were identified with first-degree heart block (prolonged PR interval) and criteria for left ventricular hypertrophy (RV6+SV1=37mm). Two demonstrated mild ST depression (1 to 2mm depression) over the left precordial leads reflecting possible myocardial ischemia.

### Arrhythmias

**FIGURE 1.**
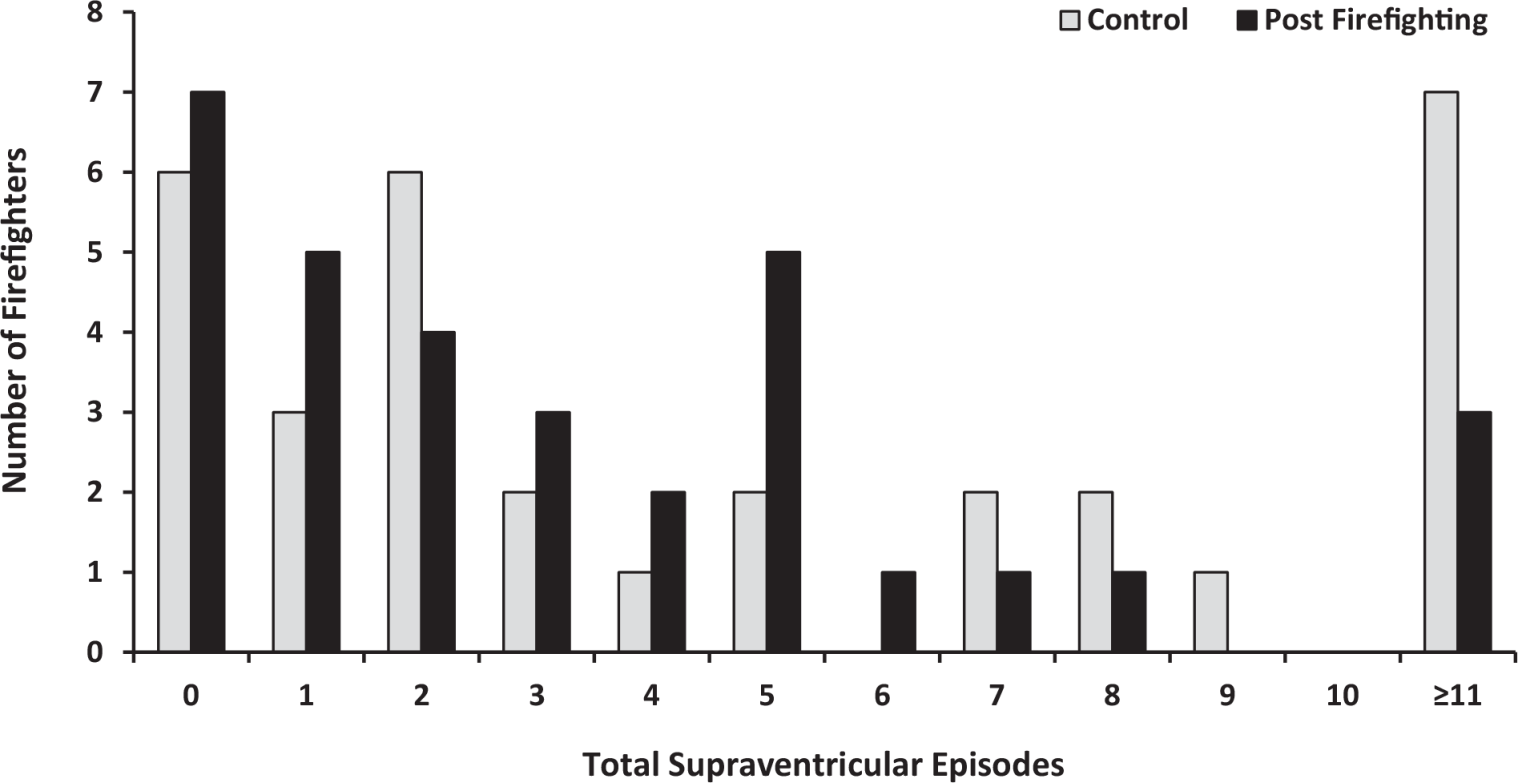
Distribution of ECG-detected supraventricular ectopy in firefighters during the 12-hour control and post-firefighting assessment periods. Note: the more than or equal to 11 category includes firefighters with 13, 17, 19, 20, 33, 42, and 51 total episodes during control and firefighters with 11, 27, and 132 total episodes during firefighting. ECG, electrocardiographic.

**FIGURE 2.**
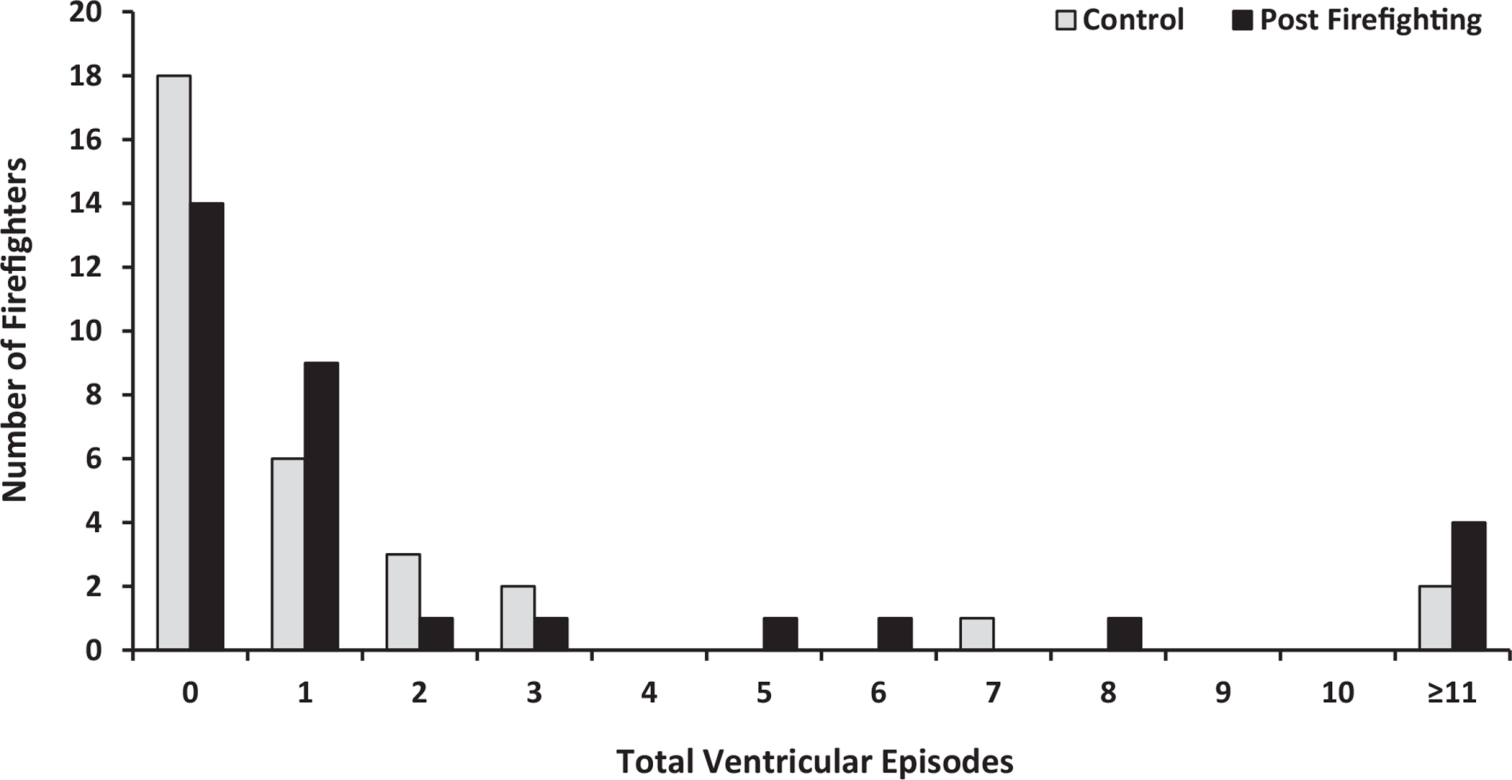
Distribution of ECG-detected ventricular ectopy in firefighters during the 12-hour control and post-firefighting assessment periods. Note: the more than and equal to 11 category includes firefighters with 16 and 54 total episodes during control and firefighters with 14, 55, 56, and 68 total episodes during firefighting. ECG, electrocardiographic.

Figures 1 and 2 present the number of firefighters who experienced various numbers of supraventricular or ventricular ectopic beats, respectively, during the 12-hour post firefighting period and during the 12-hour control period. During the 12-hour control period, supraventricular ectopy was detected in 26 firefighters. In 13 firefighters, this consisted of scattered isolated premature supraventricular beats. Thirteen of the participants displayed at least one episode of two or more beats of reciprocal supraventricular rhythm. No sustained supraventricular tachycardia was observed. Fourteen firefighters demonstrated ventricular ectopy during the 12-control period. This consisted entirely of isolated unifocal premature ventricular beats. No couplets, triplets, or runs of ventricular tachycardia were observed.

In the 12-hour period following firefighting activity, arrhythmias were recorded in 28 participants. These included 25 firefighters with supraventricular beats and 18 with ventricular ectopy. Ventricular couplets, triplets, or runs of ventricular tachycardia occurred in three firefighters.

Two firefighters displayed 11 or more episodes of ventricular ectopy during both the control and post-firefighting periods, with more episodes displayed during control than post-firefighting for one firefighter (54 vs 14) but not the other (16 vs 68). Three firefighters each had 55 or more episodes of ventricular ectopy, which consisted of isolated ventricular beats in all but one episode, detected following firefighting activity. One firefighter with 56 episodes of ventricular ectopy following firefighting also demonstrated 132 episodes of isolated supraventricular beats during the same period; during control, this same firefighter demonstrated only three episodes of isolated ventricular ectopy and no supraventricular beats.

Eleven or more supraventricular beats were detected in seven firefighters during control and three firefighters following firefighting activity, but only one firefighter who had 20 and 27 supraventricular beats during control and post-firefighting, respectively, was included in both groups. The number of supraventricular episodes detected for the more than and equal to 11 category (Fig. 1) ranged from 11 to 132 post-firefighting and from 13 to 51 during control, when 11 to 20 episodes were detected in four firefighters and more than 30 episodes were detected in three firefighters. Among the three firefighters with more than and equal to 11 supraventricular beats following firefighting, 169 of the 170 episodes detected consisted of isolated beats. During control, five firefighters displayed primarily isolated supraventricular beats, but two firefighters displayed two or more beats of supraventricular rhythm more frequently than isolated beats (22 vs 20 and 12 vs 7).

As shown in Table 2, seven participants with no ventricular arrhythmia in the control period demonstrated ventricular ectopy (six isolated, one multifocal) following simulated firefighting, whereas three participants demonstrated ventricular ectopy during the control period but not following simulated firefighting. Five firefighters who had no supraventricular beats during the control period demonstrated supraventricular ectopy following simulated firefighting; in six firefighters, supraventricular beats were detected only during the control period. No statistically significant difference existed between the number of firefighters with detected ventricular (*P*=0.34) or supraventricular (*P*=1.00) disturbances of normal cardiac rhythm between the control and post-firefighting periods.

### ST-T Segment Depression

During the control period, the ECG of one of the firefighters demonstrated episodes of possible (1–2mm depression) and definite (>2mm depression) myocardial ischemia. Following firefighting, markers of ischemia were observed to reflect possible ischemia in four firefighters and definite ischemia in two, with one of these also exhibiting several episodes of possible ischemia. The one firefighter with ischemic ST changes in the control period continued to demonstrate both possible and definite ischemia following simulated firefighting. Therefore, five participants who had normal ECGs in the control period developed indicators of myocardial ischemia (possible, *n*=4, definite *n*=1) following firefighting.

## DISCUSSION

This study revealed evidence of the onset of potentially significant arrhythmias or ECG changes typical of myocardial ischemia in the 12-hour period following a realistic bout of live-firefighting activities on a simulated fireground. Seven firefighters (20%) demonstrated the onset of ventricular ectopy while five firefighters (16%) developed new ECG findings of possible or definite myocardial ischemia. This study supports previous research that has found that firefighting may lead to significant ECG changes but extends that work by comparing results from the 12-hour period after firefighting to a control period.

Although the findings of this study are consistent with some previously published research and provides a potential mechanistic link with the increased risk of sudden cardiac events in the hours after firefighting, interpretation is challenging given the complexity of the findings and the difficulties in directly linking ECG changes with sudden cardiac events. Some have considered ECG changes during and immediately following firefighting as reflecting an etiologic link between ECG markers of ischemia and electrical instability with the augmented risk of sudden death during firefighting activities.^9–11^ It is possible that such ECG changes reflected occult underlying coronary artery disease in the ostensibly healthy firefighters in this study. However, if true, this finding is especially troubling as all participants were medically evaluated and cleared for duty within the previous 12 months. Further, none of the firefighters revealed any signs or symptoms of cardiac stress during the firefighting or the control period. Still, that such ECG responses to firefighting might serve as harbingers of sudden cardiac death is consistent with a theoretical model that poses that cardiac strain associated with the combined stressors of firefighting can cause ischemic or arrhythmogenic changes that can precipitate sudden cardiac events in fire fighters, and this is more far more likely in individuals with underlying disease.^6^

The ECG findings in this study are not dissimilar to those of previous reports. Al-Zaiti et al,^11^ for instance, described ECG changes during and for 2 hours following a live fire-fighting exercise in 42 firefighters with a mean age of 31±9 years. Eleven participants demonstrated “pathological” ST segments during firefighting (defined as greater than 0.5mm of ST depression in leads V_2_ to V_3_ or greater than 1mm in 2 or more contiguous leads for at least 5 minutes), and such changes were observed in two additional firefighters during recovery. Half of the subjects were described as having a prolonged QT interval during firefighting. However, as noted by the authors, this finding can be questioned because of the difficulty of making such a measurement during the tachycardia of exercise. Moreover, all ECG analyses were performed by a computer rather than expert interpretation. The authors concluded that “This study demonstrates that fire suppression...induces tachycardia and is associated with transient ECG changes suggestive of myocardial ischemia in firefighters at low risk for cardiovascular disease... These findings provide potential pathophysiological explanations for the high proportion of cardiovascular deaths among firefighters.”^11^

Hunter et al^9^ described electrocardiographic changes in 19 healthy male firefighters (mean age 41±7 years) before and after simulated firefighting. Compared with the control period, post-firefighting ECGs demonstrated the expected tachycardia (mean 162bpm) and a significant increase in “ST depression,” which was defined in terms of extent of depression of the J point plus 50ms on the ECG tracing. This result becomes difficult to interpret, since J point depression during tachycardia is highly common in normal healthy individuals and is considered physiologic when accompanied by a rapid upsloping ST segment.^12^ The authors concluded that “exposure to extreme heat and physical exertion during simulated fire suppression increases thrombogenicity, impairs vascular function, and causes myocardial injury in *healthy firefighters*” (italics ours).^9^

It should be noted that electrocardiographic patterns associated with myocardial ischemia are not infrequently observed during exercise testing in non-selective studies of both firefighters and the general adult population. Barnard et al^15^ performed near-maximal treadmill exercise tests on 90 random men ages 40 to 59 years from the City of Los Angeles Fire Department. Ischemic changes, defined as “at least 1mm of horizontal or down slanting depression of the ST segment observed either during exercise or in the 5 minute post exercise recovery period,” were found in nine participants (10%). A similar exercise study of insurance underwriters in the same exercise testing laboratory revealed an incidence of 8%.^16^

In these studies, too, the extent that such numbers reflect occult coronary artery disease is uncertain. In fact, it is recognized that “up to 10% of normal men and an even higher percentage of normal women may have false-positive tests (ST depressions without obstructive coronary disease).”^17^ The conclusion here is that ST changes during or after heavy work by firefighters does not necessarily imply either the presence of occult cardiovascular disease or risk for sudden death.

There is growing awareness that conditions of extended cardiac work by itself can be accompanied by appearance of biomarkers of heart stress. A rise in cardiac enzymes, left ventricular diastolic dysfunction by echocardiography, an increase in serum troponin level, and abnormal electrocardiographic findings—all markers of myocardial stress—have been reported in over half of the participants in ultra-endurance running events. For example, Herm et al^18^ reported that 9.3% of the runners in a Berlin marathon race demonstrated evidence of non-sustained ventricular tachycardia and 7.5% showed transient ST-T wave changes during the event.

The mechanistic explanation and clinical implications of these changes are not altogether clear.^19^ It is generally accepted, though, that such responses to prolonged heart work do not reflect clinically-important myocardial dysfunction nor disposition to risk for sudden cardiac death given: (a) the magnitude of change of such biomarkers is small and does not mimic that of true myocardial dysfunction (as observed in patients with myocardial infarction), (b) these changes are transient and rapidly reverse to normal (typically within 24hours), and (c) the observation of the natural “experiment” that if such changes truly reflected myocardial damage, it should have become apparent among the hundreds of thousands of participants in such endurance athletic events who, to the contrary, maintain not only their well-being but superior cardiac function.

Could the ECG changes reported in a minority of firefighters with the strenuous work of fire suppression activities fit into this explanatory category? There is, in fact, some evidence to support this conclusion. Hunter et al^9^ measured plasma levels of cardiac troponin (a marker of myocardial damage) in their study of healthy firefighters before and after a bout of simulated firefighting. Mean value doubled from 1.5 to 3.0 ng/L post firefighting simulation. The latter value was still within the normal reference range and was less than that observed in clinical setting of myocardial injury. Similar findings have been described in recent reviews of troponin changes with marathon and triathlon participation in which over half of the participants demonstrated a rise in post-race troponin concentration.^20,21^

Echocardiographic assessment of athletes immediately after ultra-endurance events of over 1-hour duration have demonstrated a “nearly ubiquitous” evidence of transient left ventricular diastolic dysfunction.^12^ Fernhall et al^22^ demonstrated similar echocardiographic findings of depressed lateral ventricular wall relaxation velocity immediately following a 3-hour period of live firefighting exercise.

In conclusion, the findings in this study support the limited research literature indicating that a minority of firefighters will demonstrate electrocardiographic findings of myocardial ischemia as well as isolated ventricular ectopy in response to a period of active fire suppression. It is not illogical to suppose that such findings reflect true myocardial stress that could impose a risk for sudden death. In fact, this logic is consistent with epidemiological evidence indicating that strenuous activity can trigger sudden cardiac events, and data documenting much higher risk of sudden cardiac events following firefighting activity compared with station duties.^1–4^ On the other hand, alternative explanations are possible. Such ECG changes could, for instance, reflect an expression of strenuous or prolonged cardiac work that, like those of endurance athletes, are transient and benign. Future research efforts to assess the frequency and “meaning” of ECG changes during fire suppression are justified to better assure the safety and health of firefighters. Additionally, research into better strategies to detect potential underlying cardiovascular disease in firefighters who are exposed to high levels of cardiac strain is also needed.

## Figures and Tables

**TABLE 1.** Baseline ECG Characteristics During Control Period (*N*=32)

Variable	Mean±SD	Abnormalities (*n*, %)
P-R interval	0.14±0.03	2 (6%)
QRS interval	0.09±0.02	1 (3%)
QTc interval	0.39±0.02	
P axis	51±15	
QRS axis	55±30	
T axis	44±17	
QRS-T angle	29±20	
Left ventricular hypertrophy		1 (3%)
SV1	8±4	
Sum of SV1 and RV6	19±6	
ST-T wave changes		2 (6%)

ECG, electrocardiographic.

**TABLE 2.** Distribution of Firefighters With ECG-Detected Ventricular and Supraventricular Ectopy During the 12-hour Control and Post-Firefighting Assessment Periods

			**Control**		
			**No (*n* [%])**	**Yes (*n* [%])**	**Total (*n* [%])**

Ventricular ectopy	Post firefighting	No	11 (78.6)	3 (21.4)	14 (100.0)
		Yes	7 (38.9)	11 (61.1)	18 (100.0)
		Total	18 (56.3)	14 (43.8)	32 (100.0)

			**Control**		
			**No (*n* [%])**	**Yes (*n* [%])**	**Total (*n* [%])**

Supraventricular ectopy	Post firefighting	No	1 (14.3)	6 (85.7)	7 (100.0)
		Yes	5 (20.0)	20 (80.0)	25 (100.0)
		Total	6 (18.8)	26 (81.3)	32 (100.0)

ECG, electrocardiographic.

## Clinical Significance:

Firefighting increase risk of ventricular arrhythmias and ST segment changes during and following 12-hours of live-fire activities. ECG’s may provide insight into risk of fatal cardiovascular events during and following firefighting.
